# Effectiveness of Different Antimicrobial Agents on Malodor Prevention in Two-Stage Dental Implants: A Double-Blinded Randomized Clinical Trial

**DOI:** 10.1055/s-0042-1747954

**Published:** 2022-07-12

**Authors:** Amirhossein Fathi, Mansour Rismanchian, Sara Nasrollahi Dezaki

**Affiliations:** 1 Dental Materials Research Center, Dental Prosthodontics Department, School of Dentistry, Isfahan University of Medical Sciences, Isfahan, Iran; 2 Dental Implants Research Center, Dental Prosthodontics Department, School of Dentistry, Isfahan University of Medical Sciences, Isfahan, Iran; 3 Dental Students Research Committee, School of Dentistry, Isfahan University of Medical Sciences, Isfahan, Iran

**Keywords:** antimicrobial agents, malodorous, two-stage dental implant systems

## Abstract

**Objective** Opening of a healing abutment in two-stage implant systems is usually followed by a bad smell. Previous studies have found that presence of bacteria in microleakages of the implant-abutment interface results in further malodor. However, studies focusing on preventive treatments for this issue are scarce. Therefore, the aim of this study is to evaluate the effectiveness of two antimicrobial agents on prevention of malodor followed by opening the healing abutments.

**Materials and Methods** Current double-blinded randomized clinical trial was performed on 51 eligible patients who were referred for their exposure surgery. They were divided equally into three parallel groups. In two groups, either chlorhexidine or tetracycline was added to the internal surface of the fixtures before screwing the healing abutments. One group did not receive any intervention. Three to 4 weeks later malodor was scored by sniffing the healing abutments immediately after uncovering them (odorless = 0/odor = 1). The three groups were then compared regarding malodor scores.

**Results** Our findings showed that malodor was more frequent in the control group (58.82%) in comparison with groups of intervention (17.65 and 23.53%). There was a statistically significant difference between malodor in patients in whom antimicrobial agents (chlorhexidine and tetracycline) were used in their implants and the control group ( *p* -value = 0.023). However, malodor in the chlorhexidine group and tetracycline group did not show any significant difference ( *p* -value = 1).

**Conclusion** Based upon the data from this study, it appears that local antimicrobial agents including chlorhexidine and tetracycline result in less malodor production within the implant-abutment interface.

**Clinical Significance** A specific type of malodor is commonly seen after opening the healing abutment of a two-stage dental implant. Not only this issue is noticed by the dentist, but also annoyed the patient. Using local antimicrobial agents in the fixtures is likely to be a simple, easily applicable solution that satisfies both patients and dentists, and eliminates the possibility of further inflammation.

## Introduction

Loss of dentition is still prevalent among societies, especially in the elderly and middle-age people. 1 It is estimated that complete edentulism is seen in 7 to 69% of adults internationally. 2 For this mean, dental implants have been used to improve function and esthetic in edentulous patients. 3 Dental implants are introduced as a treatment with a good prognosis. Most studies indicate a success rate of more than 90% for dental implants. 1 4 5 They are offered in two general types of one-stage (tissue level) and two-stage (bone level) implants. 6

Although dental implants have a good predictability, both technical and biological complications have been reported. 7 8 9 Biological complications (i.e., peri-implantitis and bone loss) may occur due to reaction against microorganisms present in oral cavity. 9 10 Beside from implant failure, oral pathogenic bacteria—mostly anaerobic—are also responsible for oral malodor, which is defined as an unpleasant odor coming out of the mouth, nose, sinuses, or pharynx. 11

Presence of pathogenic bacteria in unavoidable microleakages of implant-abutment interface in two-stage dental implant systems may cause further malodor production and peri-implantitis. 12 13 14 According to studies and clinical observations, transmucosal depth of two-stage implants is associated with anaerobic bacteria population, thereby producing malodor. 15 Several studies have demonstrated effectiveness of chlorhexidine in reducing oral bacteria. 16 17 It has also been indicated that topical administration of tetracycline results in significant reduction of oral bacteria. 18

Malodorous followed by opening of the healing abutment is usually noticed by both patients and the dentist. Patients may also ask their dentist about the possibility of a persistent malodor. However, there are few studies focusing on malodor prevention in this specific situation. Therefore, the aim of this study is to assess and compare the effectiveness of two harmless topical antimicrobial agents in prevention of the bad odor releasing after opening the healing abutments.

## Materials and Methods

### Study Design

The current study was a controlled clinical trial with “stratified randomization” and “double blinding,” which was performed on patients who were referred to the implant department of Isfahan University of Medical Sciences, Isfahan, Iran, for surgical exposure of their two-stage implants from July 2021 to September 2021. We performed our study using three equal groups, each containing 17 eligible participants.

### Sample Size Calculation

The sample size was calculated based on a similar study 17 and the following formula, which indicated that with a sample of 17 participants per group, the power of 0.8 would be obtained at 0.05 level of significance:



### Inclusion Criteria

☑ Patients who were referred for surgical exposure of their two-stage implants, and were treated with a SNUCONE implant system were included in this randomized clinical trial.
☑ All patients included had healthy periodontal conditions (less than 10% bleeding on probing) and a transmucosal depth—transmucosal depth (gingival height [GH]) evaluation was performed using a color-coded Michigan Williams periodontal probe and was measured at four points and the maximum depth was recorded—of 1 to 3 mm since these factors can affect malodorous in this study.

### Exclusion Criteria

⌧ The individuals who were either smokers or in any specific medical condition were excluded from the study.
⌧ The individuals who showed current halitosis (halitosis was assessed using an organoleptic method—the organoleptic measurement of breath was taken at a distance of 10 cm from oral cavity by a single investigator who was trained and calibrated for this task, and patients were then scored as follows: – = negative halitosis, + = positive halitosis) during our primary assessment were also excluded.
⌧ The individuals who had used antibiotics during the past 4 weeks of their visit were also excluded since it could cause bias in our results.

Using the stated criteria, 51 eligible patients were collected as samples, which were then divided into three parallel and equal study groups (group 1, group 2, and group 3). To eliminate potential bias, we engaged in a stratified randomization procedure 19 to divide the samples into three equal subgroups.

### Randomization

At first, samples were stratified (layered) into six blocks; two levels of gender (male and female) and three levels of age (less than 40, between 40 and 60, and more than 60 years old).

**Fig. 1:**
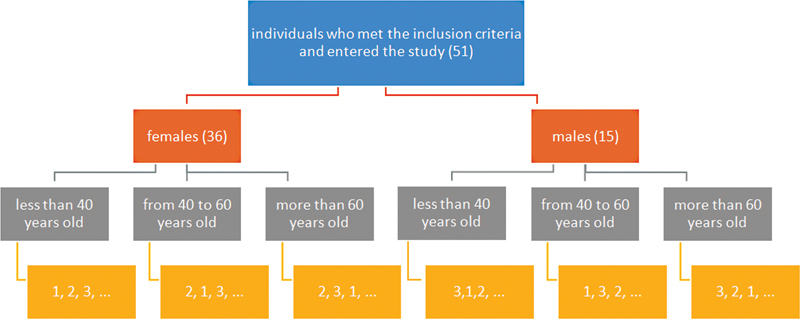
Stratified randomization procedure.

For assigning the participants within each block to one of the three study groups, six potential sequences are possible. Therefore, a sequence was given to each block randomly (i.e., the first participant of the first block enters group 1, the second participant of the first block enters group 2, the third participant of the first block enters group 3, etc.) ( Fig. 1 ).

Eventually, all layers were equally and randomly assigned into three parallel groups of 17 people. Female–male ratio was equal between the three groups and according to one-way analysis of variance, no statistically significant difference was found between age averages of the three groups ( *p* -value = 0.987).

### Intervention

In the exposure surgery session, fixtures were exposed surgically applying a flap. Then for the first group chlorhexidine 2% gel (Morvabon brand; made in Morvabon Company in Iran) and for the second group tetracycline 3% ointment (made in Hakim Company in Iran) was added to the internal surface of the fixtures with a sterile microbrush Fig. 3 ). No additional intervention was performed on the third group. Eventually, the healing abutments were screwed into the implants hand-tight using the screwdriver.

**Fig. 2:**
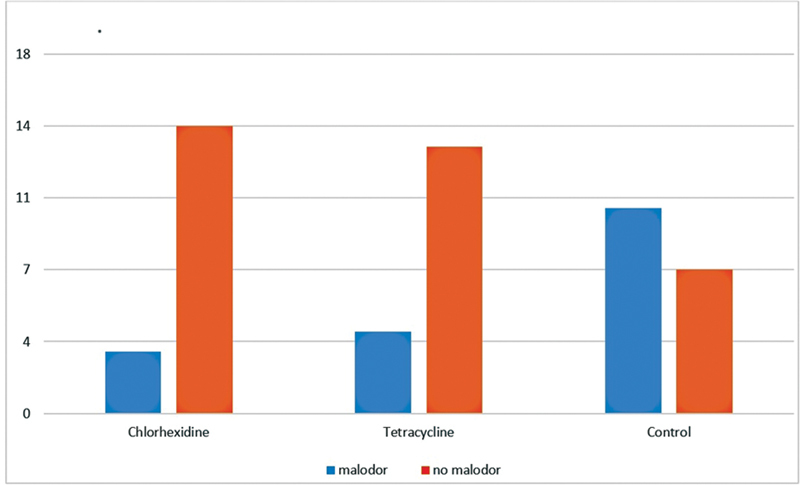
Frequency distribution of malodor within the study groups.

**Fig. 3:**
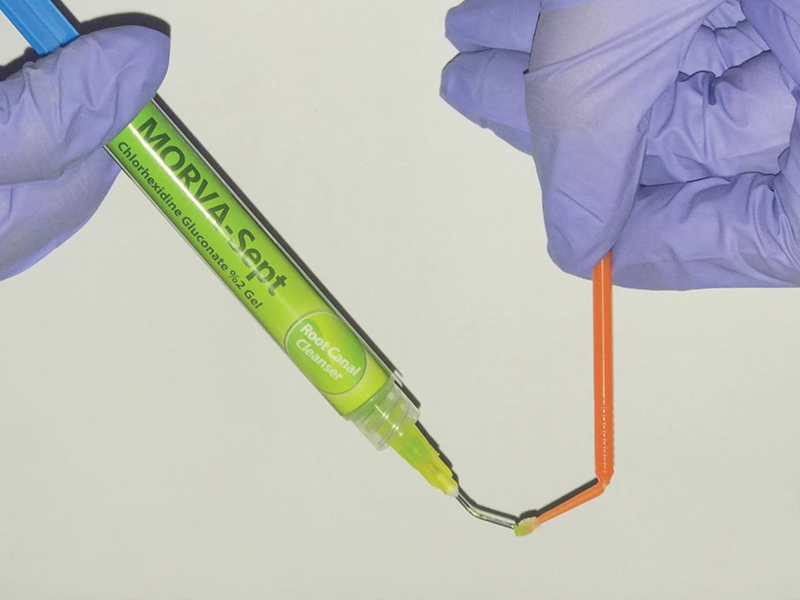
Applying chlorhexidine gel on a microbrush.

After the surgical procedure, patients were asked to brush their teeth (using toothbrush and toothpaste) and floss once a day until their next visit. They were then given postsurgery instructions and followed until their next visit.

### Measurements

Measurements were conducted 4 to 5 weeks following second-stage surgery at the first removal of the healing abutment. Patients were asked to have their breakfast meal properly early in the morning and avoid eating or drinking for 2 hours prior to measurements. Measurements included subjective odor score which was performed by a single independent observer whose functioning of olfaction was tested using The Sniffin' Sticks test. 20 Malodor was scored by sniffing the abutment immediately after uncovering it. Odor emitting from the healing abutment was scored as followed: 0 = odorless; 1 = odor.

▪ The organoleptic test (subjective assessment of odor) is not only a simple, low-cost method, but is considered to be the gold standard for odor assessment. 21 22 23
▪ The number of fixtures implanted for each patient varied from 1 to 4 fixtures. But when measuring odor, even if only one of the patient fixtures was given a score of 1, we considered point 1 for that person considering that even one smelling fixture can result in dissatisfaction.

*Blinding* : Our method was performed in three steps, each by an individual investigator:

1. The first investigator (A.F.) assessed the participants in terms of inclusion and exclusion criteria and finalized the number of samples. He performed the whole randomization process in the examination session and assigned the participants into the study groups. A code was then given to each patient. Then, he transferred the list of the participants of all three study groups to the surgeon. However, the patients were not informed which group they were participated in.
2. The surgeon (M.R.) attended the exposure surgery session and performed the intervention on the participants based on the group each were participated in. Again, patients were not informed about the exact intervention during the exposure surgery.
3. The last investigator (S.N.) attended only the measuring session (3–4 weeks after the exposure surgery when opening the healing abutments) and assessed the outcomes.

Conclusively, neither patients nor the outcome assessor was informed about the medication used for each particular patient (double-blinded).

### Statistical Analysis

Data was analyzed by IBM SPSS Statistics 24. For comparison of qualitative data between groups, the chi-square test was used. A value of *p* < 0.05 was indicated as statistically significant.

## Results

The aim of the current study was to evaluate the effectiveness of two antimicrobial agents (chlorhexidine and tetracycline) in preventing malodor associated with two-stage implants. The study sample included 51 patients who were referred to the implant department for surgical exposure of their bone-level implants. Participants were divided into three equal groups. Each group included 12 females (70.6%) and 5 men (29.4%). Baseline and clinical characteristics of the patients are reported in Table 1 .

**Table 1:** Mean and standard deviation (SD) of age and gingival height within the study groups

Group category	Age (y) Mean ± SD	Gingival height (GH) (mm) Mean ± SD
Group 1 (chlorhexidine)	55.0 ± 10.18	2.03 ± 0.495
Group 2 (tetracycline)	54.5 ± 8.78	1.99 ± 0.508
Group 3 (control)	54.7 ± 9.50	1.93 ± 0.572
Total	54.7 ± 9.31	1.98 ± 0.517

According to one-way analysis of variance, the difference between mean age and mean GH of the three groups was not statistically significant ( *p* -value = 0.987 and 0.857).

According to the chi-square test malodor frequency between the three groups was significantly different ( *p* -value = 0.023). According to Fisher's exact test there was no significant difference between malodor in chlorhexidine and tetracycline group ( *p* -value = 1). Moreover, chi-square test showed that there was a significant difference between malodor scale in the chlorhexidine group and control group ( *p* -value = 0.013) and there was also a significant difference between malodor scale in the tetracycline group and control group ( *p* -value = 0.037) Table 2 .

**Table 2:** Frequency distribution of malodor within the study groups

Scale of malodor		Study groups			Total
		Chlorhexidine	Tetracycline	Control	
No Malodor 0	Count	14	13	7	34
	Percentage	82.4%	76.5%	41.2%	66.7%
Malodor 1	Count	3	4	10	17
	Percentage	17.6%	23.5%	58.8%	33.3%
Total	Count	17	17	17	51
	Percentage	100.0%	100.0%	100.0%	100.0%

## Discussion

In this study, we aimed to evaluate the effectiveness of chlorhexidine and tetracycline on preventing malodor associated with dental implants in comparison with the control group. Our findings showed a statistically significant difference between malodor in patients whom antimicrobial agents were used in their implants and the control group. However, malodor in the chlorhexidine group and tetracycline group did not show any significant difference.

Two-stage implants contain two components: the intraosseous (fixture) and the extraosseous component (abutment or healing screw). Most mechanical and biological issues related to dental implants originate from the implant-abutment interface. 24 25 For instance, bacterial contamination occurs in the microgap between the two components both when the healing screw and when the permanent abutment is applied. When a healing screw is removed, malodorous is a result of the presence of the bacteria and their volatile sulfide compounds. 15 26 27 In 1997, Jansen et al reported that there are unavoidable microleakages at the implant-abutment connection. They suggested that bacterial colonization that occurs in the microleakages, close to the epithelial attachment, will result in further peri-implant bone resorption and implant failure. 24 Furthermore, Resende et al concluded that saliva infiltration and bacterial growth could occur in microgaps and cause inflammation and malodor. 28 Based on a recent pilot *in* *vitro* study, obtaining a complete seal against bacterial colonization at the implant-abutment interface is unlikely. 29 Scarano et al evaluated the efficacy of an antibacterial coating in the internal chamber of the implant using real-time volatile organic compound measurement technique. Based on their results, the antibacterial coating has been effective in reducing bacterial activity. 30 In the present study, chlorhexidine and tetracycline were effective in prevention of the malodorous when applied topically in the fixtures.

According to a study performed by Scarano et al, larger microgaps will result in more bacterial growth. 31 Based on previous studies, microgap size is affected by materials and fabrication methods, and significantly different microgap sizes have been reported among different implant systems. 32 33 In the current study, a single implant system was assessed considering that various systems are likely to have different sizes of microgaps. Therefore, our results may not be extensible to other implant systems.

Researchers have shown that the anaerobic bacteria located in the back of the tongue play an important role in the production of malodor, since this region is not cleaned properly. 34 35 36 It has also been reported that the bacteria responsible for malodor are mostly *Fusobacterium nucleatum* and *Porphyromonas gingivalis* . 37 38 39 There are two general ways for assessing malodor: organoleptic and instrumental technique. In organoleptic method, malodor is evaluated by the examiner's sense of smell. For instrumental measurement, various devices are used. Despite the fact that organoleptic measurement is known to be gold standard for this mean, it is associated with undeniable bias due to its subjectivity, and therefore this was a limitation of the present study. 21 22 23

For one thing, our findings provided a better understanding of a common issue with two-stage dental implants, which is particularly annoying for patients as well as dentists. We found that chlorhexidine and tetracycline were both effective in reduction of malodor when applying locally in the fixtures. Whether or not our findings can generally be used as a probable predictive treatment in this condition needs further investigation. It is also suggested for future studies to focus on identifying bacteria responsible for this issue, and use specific antibiotics based on their susceptibility.

In conclusion, based upon the data from this study, it appears that local antimicrobial agents including chlorhexidine and tetracycline result in less malodor production within the implant-abutment interface.

## References

[1] Sharma A Shrestha B Chaudhari B K Preferred source and perceived need of more information about dental implants by the undergraduate dental students of Nepal: all Nepal survey Int J Dent 2018 2018 6.794682E6 10.1155/2018/6794682 PMC5866872 29713346

[2] Petersen P E Bourgeois D Bratthall D Ogawa H Oral health information systems–towards measuring progress in oral health promotion and disease prevention Bull World Health Organ 2005 83 09 686 693 16211160 PMC2626332

[3] Rajgiri S U Dayalan M Full-mouth rehabilitation with implant-supported fixed prosthesis In J Oral Implantol Clin Res 2016 7 03 73 80

[4] Clementini M Morlupi A Canullo L Agrestini C Barlattani A Success rate of dental implants inserted in horizontal and vertical guided bone regenerated areas: a systematic review Int J Oral Maxillofac Surg 2012 41 07 847 852 22542079 10.1016/j.ijom.2012.03.016

[5] Sharma A Chaudhari B K Shrestha B Knowledge and perception about dental implants among undergraduate dental students BDJ Open 2019 5 01 1 5 30886741 10.1038/s41405-018-0009-1 PMC6418164

[6] Esposito M Grusovin M G Chew Y S Coulthard P Worthington H V Interventions for replacing missing teeth: 1- versus 2-stage implant placement Cochrane Database Syst Rev 2009 03 CD006698 19588400 10.1002/14651858.CD006698.pub2

[7] Ferreira P W Nogueira P J de Araújo Nobre M A Guedes C M Salvado F Impact of mechanical complications on success of dental implant treatments: a case–control study Eur J Dent 2022 16 01 179 187 34587636 10.1055/s-0041-1732802 PMC8890925

[8] Hanif A Qureshi S Sheikh Z Rashid H Complications in implant dentistry Eur J Dent 2017 11 01 135 140 28435381 10.4103/ejd.ejd_340_16 PMC5379828

[9] Passariello C Di Nardo D Testarelli L Inflammatory periimplant diseases and the periodontal connection question Eur J Dent 2019 13 01 119 123 31234222 10.1055/s-0039-1688525 PMC6635966

[10] Berglundh T Persson L Klinge B A systematic review of the incidence of biological and technical complications in implant dentistry reported in prospective longitudinal studies of at least 5 years J Clin Periodontol 2002 29 03 197 212, discussion 232–233 12787220 10.1034/j.1600-051x.29.s3.12.x

[11] Akaji E A Folaranmi N Ashiwaju O Halitosis: a review of the literature on its prevalence, impact and control Oral Health Prev Dent 2014 12 04 297 304 25525639 10.3290/j.ohpd.a33135

[12] Teixeira W Ribeiro R F Sato S Pedrazzi V Microleakage into and from two-stage implants: an in vitro comparative study Int J Oral Maxillofac Implants 2011 26 01 56 62 21365038

[13] Quirynen M van Steenberghe D Bacterial colonization of the internal part of two-stage implants. An in vivo study Clin Oral Implants Res 1993 4 03 158 161 8297964 10.1034/j.1600-0501.1993.040307.x

[14] Callan D P Cobb C M Williams K B DNA probe identification of bacteria colonizing internal surfaces of the implant-abutment interface: a preliminary study J Periodontol 2005 76 01 115 120 15830645 10.1902/jop.2005.76.1.115

[15] Sterer N Tamary I Katz M Weiss E Association between transmucosal depth of osseointegrated implants and malodor production Int J Oral Maxillofac Implants 2008 23 02 277 280 18548924

[16] De Boever E H Loesche W J Assessing the contribution of anaerobic microflora of the tongue to oral malodor J Am Dent Assoc 1995 126 10 1384 1393 7594010 10.14219/jada.archive.1995.0049

[17] Naveen N Suhas P G Vanishree N Vinitha M Bharath C Anushri M Effectiveness of three different oral hygiene techniques on Viridans streptococci: a randomized controlled trial J Indian Assoc Public Health Dent 2016 14 01 10

[18] Hayashida S Funahara M Sekino M The effect of tooth brushing, irrigation, and topical tetracycline administration on the reduction of oral bacteria in mechanically ventilated patients: a preliminary study BMC Oral Health 2016 16 01 67 27268137 10.1186/s12903-016-0224-x PMC4895927

[19] Kang M Ragan B G Park J-H Issues in outcomes research: an overview of randomization techniques for clinical trials J Athl Train 2008 43 02 215 221 18345348 10.4085/1062-6050-43.2.215 PMC2267325

[20] Hummel T Sekinger B Wolf S R Pauli E Kobal G ‘Sniffin’ sticks': olfactory performance assessed by the combined testing of odor identification, odor discrimination and olfactory threshold Chem Senses 1997 22 01 39 52 9056084 10.1093/chemse/22.1.39

[21] Brunner F Kurmann M Filippi A The correlation of organoleptic and instrumental halitosis measurements Schweiz Monatsschr Zahnmed 2010 120 05 402 408 20533102

[22] Falcão D P Miranda P C Almeida T FG Scalco M GDS Fregni F Amorim R FB Assessment of the accuracy of portable monitors for halitosis evaluation in subjects without malodor complaint. Are they reliable for clinical practice? J Appl Oral Sci 2017 25 05 559 565 29069154 10.1590/1678-7757-2016-0305 PMC5804393

[23] Alasqah M Khan S Elqomsan M A Gufran K Kola Z Hamza M OB Assessment of halitosis using the organoleptic method and volatile sulfur compounds monitoring J Dental Res Rev 2016 3 03 94

[24] Jansen V K Conrads G Richter E-J Microbial leakage and marginal fit of the implant-abutment interface Int J Oral Maxillofac Implants 1997 12 04 527 540 9274082

[25] Kano S C Binon P P Curtis D A A classification system to measure the implant-abutment microgap Int J Oral Maxillofac Implants 2007 22 06 879 885 18271368

[26] Assenza B Tripodi D Scarano A Bacterial leakage in implants with different implant-abutment connections: an in vitro study J Periodontol 2012 83 04 491 497 21780904 10.1902/jop.2011.110320

[27] Koutouzis T Implant-abutment connection as contributing factor to peri-implant diseases Periodontol 2000 2019 81 01 152 166 31407436 10.1111/prd.12289

[28] Resende C CD Castro C G Pereira L M Influence of the prosthetic index into Morse Taper implants on bacterial microleakage Implant Dent 2015 24 05 547 551 26068320 10.1097/ID.0000000000000284

[29] Smojver I Vuletić M Gerbl D Budimir A Sušić M Gabrić D Evaluation of antimicrobial efficacy and permeability of various sealing materials at the implant–abutment interface—a pilot in vitro study Materials (Basel) 2021 14 02 385 33466846 10.3390/ma14020385 PMC7830056

[30] Scarano A de Oliveira P S Leo L Festa F Carinci F Lorusso F Evaluation of a new antibacterial coating of the internal chamber of an implant via real time measurement of Volatile Organic Compounds (VOCs) Front Biosci (Elite Ed) 2021 13 02 216 225 34937309 10.52586/E879

[31] Scarano A Lorusso C Di Giulio C Mazzatenta A Evaluation of the sealing capability of the implant healing screw by using real time volatile organic compounds analysis: internal hexagon versus cone morse J Periodontol 2016 87 12 1492 1498 27420108 10.1902/jop.2016.160076

[32] Molinero-Mourelle P Cascos-Sanchez R Yilmaz B Effect of fabrication technique on the microgap of CAD/CAM Cobalt-chrome and zirconia abutments on a conical connection implant: an in vitro study Materials (Basel) 2021 14 09 2348 33946477 10.3390/ma14092348 PMC8125438

[33] Tribst J PM Dal Piva AMdO Ausiello P Kalman L Influence of implant-abutment contact surfaces and prosthetic screw tightening on the stress concentration, fatigue life and microgap formation: a finite element analysis Oral 2021 1 02 88 101

[34] Sacco G Campus G The treatment of periodontal disease using local oxygen-ozone Ozone Therapy 2016 1 03 45 52

[35] Rosenberg M The science of bad breath Sci Am 2002 286 04 72 79 10.1038/scientificamerican0402-72 11905111

[36] Mogilnicka I Bogucki P Ufnal M Microbiota and malodor—etiology and management Int J Mol Sci 2020 21 08 2886 32326126 10.3390/ijms21082886 PMC7215946

[37] Iatropoulos A Panis V Mela E Stefaniotis T Madianos P N Papaioannou W Changes of volatile sulphur compounds during therapy of a case series of patients with chronic periodontitis and halitosis J Clin Periodontol 2016 43 04 359 365 26824613 10.1111/jcpe.12521

[38] Tanda N Hoshikawa Y Ishida N Oral malodorous gases and oral microbiota: from halitosis to carcinogenesis J Oral Biosci 2015 57 04 175 178

[39] Suzuki N Nakano Y Watanabe T Yoneda M Hirofuji T Hanioka T Two mechanisms of oral malodor inhibition by zinc ions J Appl Oral Sci 2018 26 e20170161 29364345 10.1590/1678-7757-2017-0161 PMC5777415

